# 539 Do Not Put Burn Education on the Back Burner - Plastic Surgery in Burn Care

**DOI:** 10.1093/jbcr/irae036.173

**Published:** 2024-04-17

**Authors:** Jose Antonio Arellano, Sumaarg Pandya, Hilary Liu, Mario Alessandri-Bonetti, Tiffany Jeong, Francesco Egro

**Affiliations:** University of Pittsburgh Medical Center, Pittsburgh, PA; University of Pittsburgh Medical Center, Pittsburgh, PA; University of Pittsburgh Medical Center, Pittsburgh, PA; University of Pittsburgh Medical Center, Pittsburgh, PA; University of Pittsburgh Medical Center, Pittsburgh, PA; University of Pittsburgh Medical Center, Pittsburgh, PA

## Abstract

**Introduction:**

Burns injuries represent a leading cause of mortality and morbidity in the United States, with about 40,000 hospital admissions annually. These injuries are typically managed by burn surgeons who were trained in either plastic or general surgery. However, due to the Accreditation Council for Graduate Medical Education (ACGME)’s recent elimination of the burn surgery requirement for general surgery training, the role of plastic surgery in burn surgery care has become increasingly important. The aim of this study is to quantitively investigate the current role of plastic surgery in burn care at a single academic institution with an affiliated ABA-verified Burn Center.

**Methods:**

A retrospective cohort study was performed of burn patients admitted to UPMC Mercy, the only comprehensive ABA-verified Burn Center and Level I Trauma Center in Western PA, from January 1st, 2012, to December 31st, 2022. Data were obtained from a prospectively maintained internal database including all patients admitted for burn related health problems.

**Results:**

A total of 3843 patients were admitted for burn injuries, with 39.3% (n=1509) requiring surgery. Plastic surgeons were consulted for 17.5% (n=264) of these patients, performing a total of 658 procedures. Burn patients operated on by plastic surgeons received an average of 2.49±2.02 procedures. Most of the procedures performed by plastic surgeons involved severe and deep burns or anatomically challenging areas, such as the hand (n=362; 55.0%) and head/neck (n=165; 25.1%). These procedures involved either acute coverage of complex burn injuries or delayed reconstruction of post-burn deficits. For hand-related surgeries, 55.8% (n=202) were acute and 44.2% (n=160) were delayed reconstruction. Similarly, head and neck-related surgeries included 53.3% acute (n=88) and 46.7% (n=77) delayed reconstruction.

**Conclusions:**

Plastic surgeons play a crucial role in the acute and reconstructive care of burn patients, often performing multiple procedures for patients with injuries to anatomically complex areas such as the hand and head/neck. Since burn training is no longer required for general surgery residency, it is increasingly important that plastic surgeons be trained to perform complex burn reconstruction procedures and fill the ongoing shortage of burn specialists.

**Applicability of Research to Practice:**

Our research underscores the growing significance of plastic surgeons in burn care, particularly in light of evolving training requirements. This study emphasizes the practical need for specialized training to meet the demands of complex burn cases and addresses the current shortage of burn specialists effectively.

